# Carrying an unintended pregnancy to term and long-term maternal psychological distress: Findings from the Dutch prospective Amsterdam Born Children and their Development study

**DOI:** 10.1177/17455057231213737

**Published:** 2023-12-07

**Authors:** Wieke Y Beumer, Tessa J Roseboom, Marjette H Koot, Tanja Vrijkotte, Jenneke van Ditzhuijzen

**Affiliations:** 1 Amsterdam UMC location University of Amsterdam, Epidemiology and Data Science, Amsterdam, Netherlands; 2 Amsterdam UMC location University of Amsterdam, Obstetrics and Gynaecology, Amsterdam, Netherlands; 3 Amsterdam Reproduction and Development, Amsterdam, the Netherlands; 4 Amsterdam UMC location Vrije Universiteit Amsterdam, Public and Occupational Health, Amsterdam, Netherlands; 5 Social Policy and Public Health, Department of Interdisciplinary Social Science, Utrecht University, Utrecht, The Netherlands

**Keywords:** cohort study, family planning, maternal mental health, psychological distress, unintended pregnancy

## Abstract

**Background:**

Given the estimated high rate of unintended pregnancies, it is important to investigate long-term effects on psychological distress in women carrying an unintended pregnancy to term. However, research into associations between unintended pregnancies carried to term and psychological distress postpartum is mixed, and especially, evidence on long-term associations is scarce.

**Objective:**

To examine whether carrying an unintended pregnancy to term is associated with maternal psychological distress later in life, up to 12 years postpartum.

**Design:**

This study is based on the population-based birth cohort study ‘Amsterdam Born Children and their Development’ study, which included pregnant people in 2003 (*n* = 7784) and followed them up until 12 years postpartum.

**Methods:**

Unintended pregnancy was measured as a multidimensional construct, based on self-reported data around 16 weeks gestation on pregnancy mistiming, unwantedness and unhappiness. Symptoms of maternal psychological distress were assessed around 3 months, 5 years and 12 years postpartum using multiple questionnaires measuring symptoms of depression, anxiety and stress. Multiple structural equation modelling models were analysed, examining the associations between dimensions of unintended pregnancy and maternal psychological distress per time point, while controlling for important co-occurring risks.

**Results:**

Pregnancy mistiming and unhappiness were significant predictors of more maternal psychological distress around 3 months postpartum. Around 5 years postpartum, only pregnancy mistiming was positively associated with maternal psychological distress. Dimensions of unintended pregnancy were no longer associated with maternal psychological distress around 12 years postpartum. Strikingly, antenatal psychological distress was a much stronger predictor of maternal psychological distress than pregnancy intention dimensions.

**Conclusion:**

Those who carried a more unintended pregnancy to term reported more symptoms of psychological distress at 3 months and 5 years postpartum. People carrying an unintended pregnancy to term may benefit from extra support, not because of the pregnancy intentions per se, but because they may be related to antenatal psychological distress.

## Plain Language Summary

Carrying an unintended pregnancy to term and maternal psychological distress over time

Every unintended pregnancy is different, like every person is different. Nevertheless, carrying an unintended pregnancy to term might be stressful, that might impact mental health (e.g. depression or anxiety) of people carrying an unintended pregnancy to term. Research into long-term effects of carrying an unintended pregnancy to term on maternal mental health is scarce. In this study, we investigated effects of carrying an unintended pregnancy to term on maternal mental health up to 12 years postpartum. We used data from 7784 pregnant people living in Amsterdam, who participated in the Amsterdam Born Children and their Development (ABCD) study in 2003. Participants were followed up to 12 years postpartum. During pregnancy, participants answered questions about pregnancy mistiming (did the pregnancy happen at the right time), unwantedness (did they want to become pregnant) and unhappiness (how did they feel when they found out they were pregnant). We investigated these ‘dimensions’ of unintended pregnancy separately, to grasp to complexity of unintended pregnancy. Furthermore, participants answered multiple questions about experienced symptoms of depressions and anxiety around 3 months, 5 years and 12 years postpartum. People, who reported that their pregnancy was more mistimed, reported more mental health problems up to 5 years postpartum. Furthermore, people who reported more unhappiness with their pregnancy, reported more mental health problems around 3 months postpartum. People with an unintended pregnancy reported no longer more mental health problems around 12 years after birth, compared to people with more intended pregnancy. Strikingly, the mental health of people during pregnancy was more important for their mental health later in life, compared to their unintended pregnancy. Thus, the (emotional) circumstances around the pregnancy might be more influential for mental health later in life, compared to their pregnancy intentions. People carrying an unintended pregnancy to term may benefit from extra support, tailored to their individual needs and circumstances. Nevertheless, our results also showed that people are also resilient to deal with the many events and challenges faced during the periods after birth, since the effect of unintended pregnancy on maternal mental health disappeared over time.

## Introduction

When someone finds out she is unexpectedly pregnant, she is not always on a pink cloud. It is estimated that 28% of Dutch pregnancies are unintended, around 53% of those are carried to term.^1^ Given the estimated high rate of unintended pregnancies and its potential health impact on both women, their partners and children, it is important to investigate long-term psychological distress in women who carried an unintended pregnancy to term. Throughout this study, we will sometimes use the terms woman and women for practical reasons. This includes all persons with a uterus who do, or do not, identify as female.

Psychological effects of unintended pregnancy have been widely studied in women having abortions,^2–4^ but very few studies focused on women who carried an unintended pregnancy to term. Available previous studies investigating short-term effects of unintended pregnancies that were carried to term on maternal mental health found mixed results.^5,6^ While some studies did find that carrying an unintended pregnancy to term was associated with a risk of maternal mental health problems,^5,7–9^ others did not.^10–12^ These mixed findings might first be explained by different categorizing approaches of unintended pregnancy in previous studies. Second, the context in which unintended pregnancies are carried to term may be very different in these studies. In some countries, women have fewer options to terminate the pregnancy than in others, which may lead to different results as well. Third, the use of different measures of mental health (depression, anxiety, mood disorders, and so on), different measurement moments and the different comparison groups that were used might also explain differences in results.

Research on the long-term risks for women who carried an unintended pregnancy to term is scarce.^13^ Studies on associations between unintended pregnancies carried to term and maternal mental health over time have mostly been done in countries with less available abortion care, such as the United States.^5,14,15^ Women being denied an abortion reported higher anxiety symptoms and perceived distress compared with women who received an abortion, although the two groups converged over time.^14,16^ However, the situation might be different for women who have not been denied an abortion, but chose to carry the unintended pregnancy to term. This situation might be more prevalent in countries with fairly liberal abortion laws, such as the Netherlands,^17,18^ but remains unstudied up to now.

The question remains to what extent the decision of carrying an unintended pregnancy to term is a *cause* for mental health adversities, or whether there are other underlying mechanisms influencing both. While previous studies indicated that women with unintended pregnancies more often have lower SES, are younger and are unmarried than women with intended pregnancies,^19–21^ these socioeconomic and demographic variables have been associated with a higher risk for psychological distress in the general population as well.^22,23^ Furthermore, women who have been sexually and/or physically abused have a higher risk for mental disorders, but also for having an abortion.^24,25^ Thus, in this study, we adequately controlled for these co-occurring risk factors in the analyses.

Pregnancy intentions are a complex construct with a lot of factors involved.^26,27^ Women might experience conflicting attitudes and emotions towards their pregnancy or fail to form explicit pregnancy intentions.^28,29^ Consequently, dichotomizing unintended pregnancy (being either intended or unintended) might lead to oversimplification.^28^ Thus, in this study, we tried to grasp the complexity of unintended pregnancy by taking a multidimensional approach, based on the extent of pregnancy mistiming and unwantedness, and unhappiness with the pregnancy. These are in line with the key factors of pregnancy intentions pointed out by Santelli et al.^26^

The aim of this study is to examine associations between unintended pregnancy and maternal psychological distress over a longer time span (up to 12 years postpartum), while taking co-occurring factors into account. This is investigated in a large-scale population-based birth cohort in an abortion liberal context (the Netherlands), while considering the complexity of pregnancy intentions by using a multidimensional approach towards the concept of unintended pregnancies.

## Methods

### Participants and procedure

This study is part of the ABCD study.^30^ The broad aim of the ABCD study was to study the health and development of children, and their families born in Amsterdam, the Netherlands. For this ongoing population-based birth cohort study, 12,373 pregnant women living in Amsterdam between 2003–2004 were invited to participate during their first obstetric care visit.^30^ Information about the study was given by an obstetric caregiver. Two weeks later (around 16 weeks gestation), all women subsequently received an informed consent form and the first questionnaire at their home address.^31^ Women who wanted to participate signed the informed consent form filled out the questionnaire and send it back to the data collectors. Furthermore, at each subsequent data collection point, signed informed consent was obtained in the same manner.

**Figure 1.**
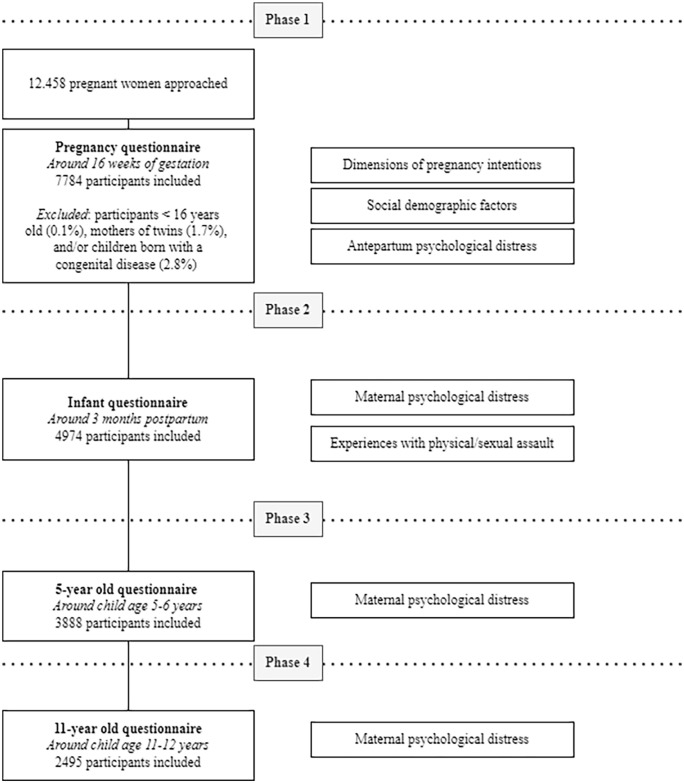
Structure of the Amsterdam Born Children and their Development study.

Of the pregnant women approached, 8266 participated in the study (response rate 67%), with selection bias being reduced to a minimum.^32^ Inclusion criteria were being pregnant currently and living in Amsterdam. Questionnaires were available in Dutch, Turkish, Arabic and English.^30^ In the current study, women who gave birth to twins (*n* = 135) and/or children with major congenital diseases (*n* = 162) were excluded. Furthermore, only participants over 16 were included in the current sample. This resulted in a final sample size of 7784 pregnant women (Figure 1).

A post hoc statistical power analysis for testing a covariance structure model using RMSEA was conducted,^33^ estimating power for a given RMSEA (null RMSEA = .05 and alternative RMSEA = .1), sample size (*n* = 7784) and an alpha of .05. Results indicated that the sample size was sufficient to answer our research questions considering the amount of parameters in the model (power >.9).^34^

Data were anonymously derived from four measurement waves in this study: (1) antepartum (around 16 weeks gestation), (2) 13 weeks postpartum (during infancy), (3) 5–6 years postpartum (start of primary school) and (4) 11–12 years postpartum (past year of primary school) (Figure 1). All measurements consisted of posted, self-reported questionnaires that were filled out at home. In each questionnaire, it was clearly stated that data were processed confidential. Filling out confidential psychological health questions at home minimized social desirability bias.^35^ For more detailed information on the study design, see Van Eijsden et al.^30^ In this study, the Strengthening the Reporting of Observational Studies in Epidemiology (STROBE) guidelines for cohort studies were followed.

### Outcome measures

Due to over-time changes in the ABCD cohort study design, psychological distress was assessed with different instruments measuring symptoms of anxiety and depression. During the first and second measurements, the State-Trait Anxiety Inventory State form (STAI-S)^36,37^ and the Center for Epidemiological Studies Depression (CES-D)^38,39^ scale were used to measure anxiety and depression, respectively. During the third and fourth measurements, the Depression Anxiety Stress Scale (DASS-21)^40,41^ was used to measure depression and anxiety concurrently, because this instrument was less time consuming and its psychometric qualities were comparable. All three instruments have been demonstrated to be good predictors of clinical anxiety and depression, and the STAI and CES-D correlate moderately to strongly with the DASS-21 anxiety or depression subscales.^42,43^ Hence, it is assumed that the use of different instruments might not have influenced the estimation of symptoms of depression and anxiety in this study.

#### Maternal psychological distress around 3 months postpartum

Around 3 months postpartum, depressive symptoms were measured with the reliable and validated Dutch version of the CES-D,^38,39^ measuring the frequency of depressive symptoms experienced over the preceding week. In line with results of previous studies into the robustness and suitability of the CES-D,^44^ depressive symptoms were modelled based on a 14-item three-factor structure: (1) negative affect, (2) anhedonia and (3) somatic symptoms. These are congruent with current diagnostic criteria for depression.^45^ Items were rated on a 4-point Likert-type scale ranging from 0 (*rarely or none of the time*) to 3 (*most of the time*).

Furthermore, symptoms of state anxiety were assessed with the reliable and validated Dutch version of the 20-item STAI-S.^36,37^ The STAI-S measured experienced temporarily anxiety over the preceding week. Items were rated on a 4-point Likert-type scale ranging from 0 (*rarely or none of the time*) to 3 (*most of the time*). A latent factor ‘anxiety’ was modelled based on two second-order latent variables: (1) positive-state anxiety and (2) negative-state anxiety. This latent structure was substantiated by previous studies into the factorial structure of the STAI-S^46,47^ and further confirmed by the results from the confirmatory factor analysis in this study.

#### Maternal psychological distress around 5–6 years postpartum

Around 5–6 years postpartum, maternal psychological distress was measured with the reliable and validated Dutch version of the DASS-21. The DASS-21 measured the experience of symptoms of depression, anxiety and stress over the preceding week on a 4-point Likert-type scale ranging from 0 (*never or rarely*) to 3 (*very often*). Psychological distress was explained by three first-order latent variables, representing the three subscales of the DASS-21 (depression, anxiety and stress, 7 items per subscale).^41^

#### Maternal psychological distress around 11–12 years postpartum

Around 11–12 years postpartum symptoms of psychological distress in the preceding week were measured using the DASS-21. Again, psychological distress was explained by three first-order latent factors, representing the subscales of the DASS-21 (depression, anxiety and stress, 7 items per subscale).^41^

### Predictor variables (antepartum, around 16 weeks gestation)

#### Pregnancy intentions

Pregnancy intentions were measured as a multidimensional construct, based on self-reported data on the extent of pregnancy mistiming, unwantedness and unhappiness. Dimensions of unintended pregnancy were measured with an item each: ‘*I did not want to be pregnant (anymore*)’ (unwantedness), ‘*This pregnancy happened too soon*’ (mistiming) and *‘I am happy to be pregnant’* (unhappiness; recoded). Items were rated on a 4-point Likert-type scale ranging from 0 (*definitely not true*) to 3 (*very true*), with a higher score indicating more unintendedness.

#### Co-occurring risks

Different co-occurring risks were taken into account. First, sociodemographic characteristics were obtained. Regarding maternal characteristics, we controlled for maternal age, ethnicity (being born in the Netherlands; yes/no), educational level (measured in years of educational attainment after primary school), having a paid job (yes/no) and being a single parent during pregnancy (yes/no). Furthermore, we controlled for the sex of the child (boy/girl) and the number of children already present in the family. Second, maternal experience with sexual and physical assault was assessed with two questions. Due to low prevalence, it was combined into one variable (experience with sexual and/or physical abuse, yes/no). Third, we controlled for maternal antepartum psychological distress psychological with the validated Dutch versions of the CES-D for depressive symptoms and the STAI-S for state anxiety.^36,37^ A total score was calculated for depression and anxiety each, with a higher score indicating more symptoms.

### Statistical analyses

Sample characteristics were obtained for the co-occurring risk factors. Furthermore, correlations between variables of interest were obtained. To examine whether nonresponse was selective, women with missing data on one or more of the follow-up measurement phases were compared with women who completed all measurement waves.

Statistical analyses were performed in Rstudio.^48^ Missing data were handled using Full Information Maximum Likelihood (FIML) estimation in RStudio, providing more reliable results compared with list-wise deletion^49^ and similar results to other multiple imputation methods.^50^ The *p* values less than 0.05 were considered statistically significant.

Associations between unintended pregnancy and psychological distress were investigated using Structural Equation Modelling (SEM) with the lavaan package.^51^ In a SEM path model, each estimated standardized regression coefficient can be used to interpret the unique variance of each predictor, while controlling for the effects of the other predictors.^52^ Models were considered to have a good fit when the Root Mean Square Error of Approximation (RMSEA) was smaller than .05, and adequate fit when the Comparative Fit Index (CFI) was larger than .90.^53–56^ To account for the non-normality in the data, maximum likelihood with robust standard errors (MLR) was used.^57^

**Figure 2.**
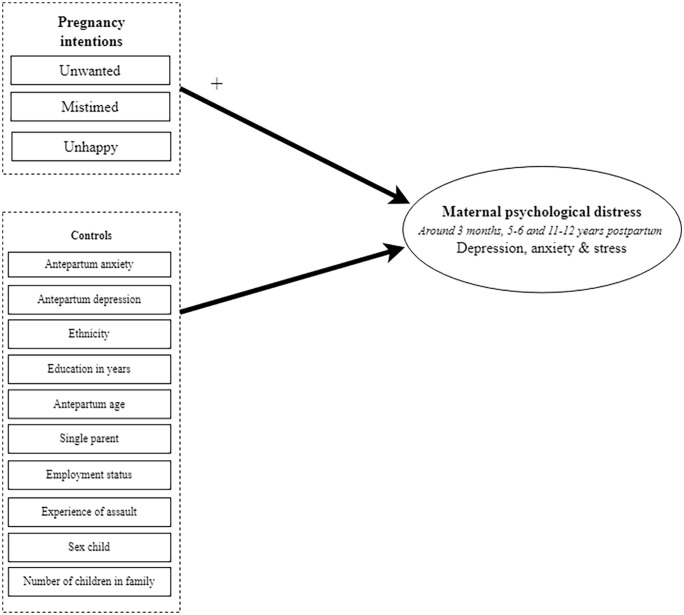
Research model.

A path model was estimated per timespan ((1) 3 months postpartum, (2) 5–6 years postpartum and (3) 11–12 years postpartum). In the model estimating the effects on psychological distress around 3 months postpartum, a model was estimated for state anxiety and depressive symptoms separately, since they were derived from different inventories and hence items overlapped too much to estimate a single model for both latent constructs. In line with recommendations from previous studies, state anxiety was modelled as a latent construct explained by a positive and negative factor.^47^ Furthermore, depression was modelled as a latent construct explained by three factors (anhedonia, negative affect and somatic symptoms).^44^ In the models estimating the effects around 5–6 years and 11–12 years postpartum, one model was estimated for psychological distress in total, explained by three factors (depression, anxiety and distress) in line with previous studies.^41^ In each model, the dimensions of unintended pregnancy were added as separate exogenous variables: (1) mistiming, (2) unwantedness and (3) unhappiness with the pregnancy. Furthermore, we controlled for maternal antepartum depression and anxiety, experience with abuse and sociodemographic variables by adding them as predictors in each model (Figure 2).

### Nonresponse analyses

Participants who were lost to follow-up (40.0% dropped out after phase 1, 59.0% after phase 2% and 69.8% after phase 3) were compared with those who responded to each measurement (*n* *=* 2035). Compared with responders, dropouts were younger and more likely to be a single parent, and unemployed. Furthermore, they more often had a migrant background and less years of education (Table 1).

**Table 1.** Characteristics of responders and nonresponders (antepartum, around 16 weeks gestation).

	Complete responders	Dropped out after phase 1	Dropped out after phase 2	Dropped out after phase 3	Dropped out but returned	
	*n* = 2035	*n* = 2192	*n* = 1454	*n* = 1300	*n* = 803	
	*M* (*SD*)	*M* (*SD*)	*M* (*SD*)	*M* (*SD*)	*M* (*SD*)	
Unintended pregnancy dimensions						
Unhappy	0.01 (0.09)	0.02 (0.17)	0.01 (0.10)	0.02 (0.10)	0.02 (0.15)	*F* = 5.07*
Unwanted	0.05 (0.27)	0.14 (0.43)	0.12 (0.39)	0.07 (0.31)	0.10 (0.36)	*F* = 18.65*
Mistimed	0.58 (0.71)	0.92 (0.90)	0.71 (0.84)	0.71 (0.81)	0.77 (0.81)	*F* = 46.31*
Baseline psychological distress						
Anxiety	35.30 (9.18)	41.03 (10.27)	38.48 (10.40)	38.07 (10.35)	39.36 (10.22)	*F* = 86.65*
Depression	10.43 (7.12)	14.45 (9.12)	12.90 (8.93)	12.6 (8.66)	13.40 (8.88)	*F* = 60.33*
Social demographics						
Age	32.56 (4.02)	29.14 (5.80)	30.18 (5.36)	31.23 (4.90)	30.61 (4.96)	*F* = 127.96*
Years of education	10.66 (3.18)	6.67 (4.18)	8.52 (3.88)	9.00 (3.90)	8.61 (4.11)	*F* = 287.31*
	*N* (%)	*N* (%)	*N* (%)	*N* (%)	*N* (%)	
Ethnicity						*χ* = 896.4*
Dutch	1708 (83.9)	875 (40.0)	842 (57.9)	890 (68.5)	491 (61.3)	
Non-Dutch	327 (16.1)	1311 (60.0)	612 (42.1)	409 (31.5)	310 (38.7)	
Employed						*χ* = 708.1*
Yes	1774 (87.6)	1105 (50.7)	997 (68.9)	974 (75.8)	572 (71.9)	
No	252 (12.4)	1074 (49.3)	449 (31.1)	311 (24.2)	223 (28.1)	
Single parent						*χ* = 36.1*
Yes	26 (1.3)	93 (4.3)	45 (3.1)	30 (2.3)	23 (2.9)	
No	2005 (9)	2087 (95.7)	1408 (96.9)	1267 (97.7)	803 (97.1)	

*M*: Mean; *SD*: standard deviation.

* *p* < .001.

## Results

### Descriptive analyses

Participants were between 16–46 years during the first measurement (antepartum). Most participants indicated that their pregnancy was much wanted but a bit mistimed, and that they were very happy with their pregnancy. Other participant characteristics are described in Table 2.

**Table 2.** Participant characteristics.

Age (*M, SD*)	
Years	30.73 (5.22)
Educational attainment (*M, SD*)	
Years	8.65 (4.10)
Ethnicity (*n*, %)	
Dutch	4806 (61.7)
Non-Dutch	2968 (38.1)
Missing	10 (0.1)
Employed (*n*, %)	
Yes	5422 (69.7)
No	2309 (29.7)
Missing	53 (0.7)
Single parent (*n*, %)	
Yes	217 (2.8)
No	7547 (97.0)
Missing	20 (0.3)
Experience of assault^a^ (*n*, %)	
Yes	71 (0.9)
No	4907 (63.0)
Missing	2806 (36.1)
Parity (*n*, %)	
Nulliparous	3174 (40.8)
Multiparous	4605 (59.2)
Missing	5 (0.0)
Sex child (*n*, %)	
Boy	3908 (50.2)
Girl	3846 (49.4)
Missing	30 (0.4)
State anxiety symptoms (*M, SD*)	
STAI score	38.35 (10.25)
Depressive symptoms (*M, SD*)	
CES-D score	12.67 (8.6)
Pregnancy unhappiness^b^ (*n*, %) *I am happy to be pregnant*	
*Completely true*	6279 (80.7)
*True*	1389 (17.8)
*Not true*	70 (0.9)
*Completely not true*	14 (0.2)
Missing	32 (0.4)
Pregnancy unwantedness^b^ (*n*, %) *I did not wanted to become pregnant (anymore)*	
*Completely true*	162 (2.1)
*True*	419 (5.4)
*Not true*	1548 (20.0)
*Completely not true*	5623 (72.2)
Missing	32 (0.4)
Pregnancy mistiming^b^ (*n*, %) *This pregnancy came too soon*	
*Completely true*	335 (4.3)
*True*	885 (11.4)
*Not true*	2904 (37.3)
*Completely not true*	3350 (43.0)
Missing	110 (1.4)

*M*: mean; *SD*: standard deviation; STAI: State-Trait Anxiety Inventory; CES-D: Center for Epidemiological Studies Depression.

a Assault was assessed as experience with sexual and/or physical assault during pregnancy.

b Dimensions of unintended pregnancy were measured on a 4-point Likert scale (0–3), with a higher score indicating more unintendedness.

With regard to correlations between our variables of interest, results indicated that on average, women who received less years of education were younger, not Dutch-born, did not have a job or were a single parent reported more pregnancy mistiming and unwantedness. However, no differences were found in happiness with the pregnancy. Furthermore, maternal psychological distress at baseline (both depression and anxiety) were positively correlated to the dimensions of unintended pregnancy, indicating that women experiencing more psychological distress antepartum also report more unintended pregnancies (Table 3).

**Table 3.** Bivariate correlations between variables of interest.

	1	2	3	4	5	6	7	8	9	10	11	12	13	14	15
1. Unhappy with pregnancy	–	.38**	.35**	.26**	.23**	.00	−.11**	−.14**	−.07**	.13**	.05*	.19**	.19**	.13**	.09**
2. Unwanted pregnancy		–	.42**	.22**	.20**	−.06**	−.25**	−.25**	−.11**	.26**	.04*	.16**	.16**	.10**	.06*
3. Mistimed pregnancy			–	.26**	.25**	−.17**	−.19**	−.23**	−.09**	.21**	.07**	.19**	.20**	.14**	.11**
4. Antepartum anxiety (STAI)^a^				–	.87**	−.18**	−.26**	−.23**	−.09**	.22**	.10**	.56**	.51**	.36**	.33**
5. Antepartum depression (CES-D)^b^					–	−.18**	−.22**	−.23**	−.10**	.19**	.12**	.51**	.54**	.37**	.34**
6. Maternal age						–	.33**	.26**	.04**	−.16**	−.08**	−.09**	−.08**	−.06**	−.01
7. Years of education							–	.44**	.12**	−.42**	−.10**	−.18**	−.17**	−.18**	−.09**
8. Employed								–	.10**	−.43**	−.04*	−.19**	−.21**	−.11**	−.11**
9. In a relationship									−	−.05**	−.10**	−.04*	−.07**	−.03	−.01
10. Non-Dutch ethnicity										−	.04*	.20**	.19**	.14**	.12**
11. Experience of assault^c^											−	.10**	.11**	.09**	.03
12. Anxiety 3 months postpartum (STAI)^a^												−	.88**	.38**	.35**
13. Depression 3 months postpartum (CES-D)^b^													−	.37**	.36**
14. Psychological distress 5–6 years postpartum (DASS-21)^d^														−	.45**
15. Psychological distress 11–12 years postpartum (DASS-21)^d^															–

STAI: State-Trait Anxiety Inventory; CES-D: Center for Epidemiological Studies Depression; DASS: Depression Anxiety Stress Scale.

a Total scores of the STAI.

b Total scores of the CES-D.

c Assault was assessed as experience with sexual and/or physical assault during pregnancy.

d Total scores of the DASS-21.

* *p* < .01; **p < .001.

### Over-time associations between unintended pregnancy and maternal psychological distress

#### Maternal psychological distress around 3 months postpartum

With regard to results on psychological distress around 3 months postpartum, the hypothesized anxiety path model showed an adequate fit, RMSEA = .043 and CFI = .902. While controlling for antepartum background variables, baseline psychological distress and other dimensions of unintended pregnancy, pregnancy mistiming and unhappiness were significant predictors of maternal anxiety levels around 3 months postpartum (β = .03, *p* *=* .050; β = .04, *p* *=* .007, respectively). Effect sizes are small^58^ due to the large number of predictors in the model. In contrast, pregnancy unwantedness did not significantly predict anxiety levels around 3 months postpartum (Table 4). Furthermore, antepartum symptoms of depression and anxiety were the strongest predictors of symptoms of anxiety around 3 months postpartum (β = .08, *p* *=* .013; β = .50, *p* *<* .001, respectively), indicating a, respectively, small and large effect size.^58^

**Table 4.** Structural equation modelling regression results of the associations between predictor variables and maternal psychological distress around 3 months postpartum.

	*b*	SE_*b*_	β	CI	*z* score	*p*
Outcome: Depression^a^						
Unintended pregnancy dimensions						
Unwanted	−0.01	0.01	−.01	[−0.02, 0.01]	−0.64	.522
Mistimed	0.01	0.01	.04	[0.00, 0.03]	2.16	.031
Unhappy	0.03	0.01	.05	[0.01, 0.06]	2.74	.009
Controls						
Antepartum depression^a^	0.01	0.00	.37	[0.01, 0.02]	9.94	.000
Antepartum anxiety^b^	0.01	0.00	.19	[0.00, 0.01	6.08	.000
Experience with sexual/physical abuse (yes = 1)	0.15	0.05	.06	[0.05, 0.26]	2.83	.007
Years of education after primary school	−0.00	0.00	−.01	[−0.00, 0.00]	−0.29	.772
Non-Dutch ethnicity (yes = 1)	0.04	0.01	.06	[0.02, 0.06]	3.47	.001
Age	0.00	0.00	.06	[0.00, 0.01]	3.16	.002
Being single during pregnancy (yes = 1)	0.00	0.04	.00	[−0.07, 0.07]	0.10	.924
Having a job (yes = 1)	−0.05	0.01	−.07	[−0.07, −0.02]	−3.67	.000
Sex child (girl = 1)	−0.01	0.01	−.01	[−0.02, 0.01]	−0.74	.463
Number of children in the family	−0.00	0.00	−.01	[−0.01, 0.01]	−0.31	.753
Outcome: Anxiety^b^						
Unintended pregnancy dimensions						
Unwanted	0.00	0.01	.00	[−0.02, 0.02]	0.25	.806
Mistimed	0.02	0.01	.03	[−0.00, 0.03]	1.94	.050
Unhappy	0.04	0.02	.04	[0.01, 0.07]	2.71	.007
Controls						
Antepartum depression^a^	0.00	0.00	.08	[0.00, 0.01]	2.49	.013
Antepartum anxiety^b^	0.02	0.00	.50	[0.02, 0.02]	15.30	.000
Experience with sexual/physical abuse (yes = 1)	0.15	0.06	.04	[0.04, 0.26]	2.59	.010
Years of education after primary school	−0.00	0.00	−.03	[−0.00, 0.01]	−1.99	.047
Non-Dutch ethnicity (yes = 1)	0.07	0.01	.08	[0.04, 0.10]	4.73	.000
Age	0.00	0.00	.03	[−0.00, 0.01]	1.85	.064
Being single during pregnancy (yes = 1)	0.04	0.04	.02	[−0.04, 0.12]	0.93	.351
Having a job (yes = 1)	−0.06	0.02	−.07	[0.01, −0.03]	−3.80	.000
Sex child (girl = 1)	−0.01	0.01	−.02	[−.04, 0.01]	−1.32	.189
Number of children in the family	0.01	0.01	.02	[−0.01, 0.01]	1.02	.306

SE: standard error; CI: confidence interval.

a Depression was measured with the CES-D.

b Anxiety was measured with the STAI-S.

Furthermore, the hypothesized depression path model was well-fitted, RMSEA = .032 and CFI = .928. In line with results from the anxiety model, pregnancy mistiming and unhappiness were significant predictors of maternal depression levels around 3 months postpartum (β = .04, *p* = .031; β = .05, *p* *=* .006, respectively), while controlling for antepartum background variables and other dimensions of unintended pregnancy. Effect sizes are small.^58^ Pregnancy unwantedness did not significantly predict depression levels around 3 months postpartum (Table 4). Furthermore, antepartum symptoms of depression and anxiety were the strongest predictors of symptoms of depression around 3 months postpartum (β = .37, *p* *<* .001; β = .19, *p* *<* .001, respectively), indicating a, respectively, medium and small effect size.^58^

#### Maternal psychological distress around 5–6 years postpartum

The hypothesized path model estimating associations between pregnancy intentions and psychological distress levels around 5–6 years postpartum showed a good fit, RMSEA = .026 and CFI = .921. While controlling for all other variables in the model, pregnancy mistiming was a significant predictor of maternal psychological distress levels around 5–6 years postpartum (β = .05, *p* *=* .040). However, pregnancy unwantedness and unhappiness did not significantly predict psychological distress levels around 5–6 years postpartum (Table 5). Furthermore, antepartum symptoms of depression and anxiety were the strongest predictors of symptoms of psychological distress around 5–6 years postpartum (β = .26, *p* < .001; β = .14, *p* *=* .001, respectively), although these effect sizes are small.^58^

**Table 5.** Structural equation modelling regression results of the associations between predictor variables and psychological distress around 5–6 years postpartum.

	*b*	SE_*b*_	β	CI	*z* score	*p*
Unintended pregnancy dimensions						
Unwanted	−0.00	0.01	−.01	[−0.01, 0.01]	−0.56	.574
Mistimed	0.01	0.00	.05	[0.00, 0.01]	2.06	.040
Unhappy	0.01	0.01	.04	[−0.00, 0.02]	1.55	.121
Controls						
Antepartum depression^a^	0.00	0.00	.26	[0.00, 0.01]	5.10	.000
Antepartum anxiety^b^	0.00	0.00	.14	[0.00, 0.00]	3.36	.001
Experience with sexual/physical abuse (yes = 1)	0.08	0.04	.07	[−0.01, 0.16]	1.78	.074
Years of education after primary school	−0.00	0.00	−.02	[−0.00, 0.00]	−0.75	.454
Non-Dutch ethnicity (yes = 1)	0.02	0.01	.08	[0.01, 0.03]	3.50	.000
Age	0.00	0.00	.01	[−0.00, 0.00]	0.35	.724
Being single during pregnancy (yes = 1)	0.01	0.02	.01	[−0.03, 0.05]	0.49	.626
Having a job (yes = 1)	−0.01	0.01	−.02	[−0.02, 0.01]	−0.91	.366
Sex child (girls = 1)	−0.00	0.00	−.01	[−0.01, 0.01]	−0.81	.421
Number of children in the family	−0.00	0.00	−.01	[−0.00, 0.00]	−0.32	.749

SE: standard error; CI: confidence interval.

a Depression was measured with the CES-D.

b Anxiety was measured with the STAI-S.

#### Maternal psychological distress around 11–12 years postpartum

The hypothesized path model estimating associations between pregnancy intentions and psychological distress levels around 11–12 years postpartum showed a good fit, RMSEA = .019 and CFI = .933. Results indicated that while controlling for all other variables in the model, the three dimensions of unintended pregnancy were no significant predictors of maternal psychological distress levels around 11–12 years postpartum (Table 6). Again, antepartum symptoms of depression and anxiety were the strongest predictors of psychological distress around 11–12 years postpartum (β = .28, *p* < .001; β = .15, *p* *=* .003, respectively), indicating small effect sizes.^58^

**Table 6.** Structural equation modelling regression results of the associations between predictor variables and psychological distress around 11–12 years postpartum.

	*b*	SE_*b*_	β	CI	*z* score	*p*
Unintended pregnancy dimensions						
Unwanted	−0.01	0.01	−.03	[−0.02, 0.01]	−1.09	.276
Mistimed	0.01	0.01	1.57	[−0.01, 0.02]	1.57	.016
Unhappy	−0.00	0.01	−.00	[−0.02, 0.02]	−0.14	.888
Controls						
Antepartum depression^a^	0.01	0.00	.28	[0.00, 0.01]	4.35	.000
Antepartum anxiety^b^	0.00	0.00	.15	[0.00, 0.00]	3.00	.003
Experience with sexual/physical abuse (yes = 1)	−0.01	0.05	−.01	[−0.10, 0.09]	−0.16	.874
Years of education after primary school	−0.00	0.00	−.03	[−0.00, 0.00]	−1.04	.296
Non-Dutch ethnicity (yes = 1)	0.02	0.01	.07	[0.00, 0.04]	2.08	.037
Age	0.00	0.00	.03	[−0.00, 0.01]	1.02	.308
Being single during pregnancy (yes = 1)	0.02	0.03	.02	[−0.04, 0.07]	0.50	.619
Having a job (yes = 1)	−0.01	0.01	−.03	[−0.03, 0.01]	−0.84	.403
Sex child (girl = 1)	−0.01	0.01	−.02	[−0.02, 0.01]	−1.13	.260
Number of children in the family	−0.01	0.01	−.02	[−0.01, 0.00]	−0.66	.507

SE: standard error; CI: confidence interval.

a Depression was measured with the CES-D.

b Anxiety was measured with the STAI-S.

## Discussion

Results of this study show that women carrying a more unintended pregnancy to term experience more symptoms of maternal psychological distress (depression, anxiety and stress) at 3 months and 5 years postpartum. However, unintended pregnancy was no longer associated with symptoms of maternal psychological distress around 12 years postpartum. Strikingly, antenatal psychological distress was a much stronger predictor of maternal psychological distress over time compared to unintended pregnancy.

This study investigated unintended pregnancy as a multidimensional construct (based on self-reported extents of pregnancy mistiming, unwantedness and unhappiness), taking its complexity into account. These dimensions are in line with the work by Santelli et al.^26^ Results of this study indicated that pregnancy mistiming was among the other dimensions of unintended pregnancy the strongest predictor of maternal psychological distress over time.

Consistent with results of previous studies,^12,59–61^ current results show that women who carried a more mistimed pregnancy to term reported more symptoms of psychological distress around 3 months and 5 years postpartum, but no longer 12 years postpartum. Although previous research into long-term effects of carrying a mistimed pregnancy to term is scarce, some studies also found that women reported diminishing levels of depression and anxiety in the first years postpartum.^62^ Moreover, previous studies into women who had access to and underwent abortions, versus women who were denied abortions, found differences in perceived distress levels soon after being denied abortions; however, those differences were no longer present on the longer term.^16^ This might explain the absence of an association between carrying a more mistimed pregnancy to term and maternal psychological distress around 12 years postpartum in this study as well. In this way, results of our study show that although women’s experiences of carrying a more mistimed pregnancy to term can have influence on maternal psychological distress over time, those women are also resilient to deal with the many events and challenges faced during these periods.

In contrast to what was found in previous studies,^5,14^ pregnancy unwantedness was not associated with maternal psychological distress at any point measured later in life. Several reasons might explain this contradiction in research findings. First, previous studies into long-term effects of carrying a (highly) unwanted pregnancy to term were mostly done in populations in which women were not legally allowed to have an abortion, or were denied one. However, in this study’s context women who carried a strongly unwanted pregnancy may have chosen to terminate the pregnancy due to the more liberal abortion context,^63^ and, therefore, not be part of the sample. Second, since participants reported on their pregnancy intentions while they were already pregnant, deliberately choosing to carry an unwanted pregnancy to term might positively influence the perception of the degree of unwantedness (i.e. reporting a pregnancy as more wanted compared with before the pregnancy).^64^ Third, social desirability might have also lead to an underestimation of pregnancy unwantedness.^64–66^ Fourth, finding an effect of pregnancy mistiming but not of unwantedness might be explained by the statistical approach of this study, since different dimensions of pregnancy intentions were added to the same statistical models. Thus, although pregnancy mistiming is the strongest predictor of symptoms of postpartum psychological distress, this might also reflect part of the effect of carrying a more unwanted pregnancy to term, since pregnancy mistiming and unwantedness were also highly correlated.

In line with results of previous studies,^60^ results of this study indicated that women who were more unhappy with their pregnancy experienced more symptoms of anxiety and depression around 3 months postpartum. Previous studies concluded that women reporting more pregnancy unhappiness felt that the costs of another baby did not weigh against other competing personal commitments or aspirations,^29^ or felt they were not prepared for maternity.^67^ This might explain the association between pregnancy unhappiness and short-term maternal psychological distress. Results of this study further indicate that this association disappears over time, since pregnancy unhappiness was no longer associated with maternal psychological distress from around 5 years postpartum. Emotions regarding a pregnancy might be less stable over time (for instance, due to other life circumstances that come along), explaining the disappearance of the effect around 5 years postpartum.

Another important result of this study is that the strongest and most consistent predictors of maternal psychological distress over time were antepartum symptoms of psychological distress. This suggests that mental health during pregnancy is an important influence on maternal health later in life, which is in line with previous studies.^68^ Hence, in line with recent recommendations from other researchers in the field,^69^ current results imply that the circumstances around the pregnancy might be more important than the actual amount of pregnancy intendedness to explain maternal psychological distress later in life.

We acknowledge several limitations of this study. First, we cannot eliminate the risk that selection bias in participants who dropped out our cohort data affected our results. Cohort studies are prone to selective dropout and hence are likely to underestimate prevalence of mental health problems.^70^ In this study, dropouts were younger and more likely to be a single parent and unemployed, compared with responders. Furthermore, they more often had a migration background and fewer years of education. Since these are all risk factors for psychological distress as well,^22,23^ it was assumed that dropouts could have possibly reported higher levels of psychological distress. Moreover, dropouts reported more unintended pregnancies and more antepartum psychological distress compared with responders. Consequently, the dropout rate might have led to an underestimation of the associations between unintended pregnancy and maternal psychological distress,^71^ and possibly the disappearance of the association between aspects of unintended pregnancy and psychological distress around 12 years postpartum. Second, we were not able to control for a history of psychological distress, which has repeatedly been found to be an important predictor of psychological distress among women who have had an abortion^2–4^ and parental (postpartum) depression.^72^ However, in this study, we controlled for psychological distress at baseline (antepartum), which might be a good proxy for mental health history due to a high consistency in reports of psychological distress over time.^73^ Third, although pregnancy intentions were measured as a multidimensional construct in line with the work by Santelli et al.^26^ some information was missing in the current cohort data. Contraceptive use might be an important dimension of pregnancy intentions, since people who (consistently) use contraceptives have higher intentions to avoid a pregnancy.^74^ Furthermore, the intentions of the partner involved in the pregnancy might be another important dimension of pregnancy intentions^28^ that also might have an influence on maternal psychological distress over time.^75,76^ Thus, future studies are advised to incorporate both the partner’s pregnancy intentions and contraceptive use when considering pregnancy intentions.

Despite these limitations, this study was the first to investigate long-term associations between dimensions of unintended pregnancy carried to term and symptoms of maternal psychological distress in an abortion liberal context. Our findings were derived from a large-scale and multi-ethnic prospective birth cohort, providing results that might be translatable to the Dutch population, and other countries with a similar abortion climate. Furthermore, in line with recommendations from previous studies,^6^ the design of this study made it possible to consider the complexity of pregnancy intentions, by taking into account different dimensions of unintended pregnancy and providing meaningful insights in different associations on maternal psychological distress. Moreover, associations were investigated while taking into account other important co-occurring risks, like socioeconomic factors (maternal age, education, relationship status, having a job and ethnicity), antepartum psychological distress and experiences with assault. Thus, reported associations are present despite other important influencers of symptoms of maternal psychological distress over time.

## Conclusion

Our study showed that pregnancy intentions and psychological distress were associated. The more unintended a pregnancy was, the higher the levels of psychological distress around 3 months and 5 years postpartum, but no longer at 12 years follow-up. Strikingly, results also showed the importance of antepartum psychological distress when explaining levels of maternal psychological distress later in life. These results implicate that women with more symptoms of antenatal psychological distress may benefit from extra support. Unintended pregnancy is a complex personal experience with a lot of other factors involved. This study, but also other studies^6^ show the importance of considering co-occurring risks when looking at psychological distress in relation to unintended pregnancy. Psychological distress may contribute to the unintendedness of a pregnancy, rather than the other way around. Future studies should explore ways to (better) support these women tailored to their individual needs, focusing on protective factors decreasing the risk on psychological distress.

## Supplemental Material

sj-docx-1-whe-10.1177_17455057231213737 – Supplemental material for Carrying an unintended pregnancy to term and long-term maternal psychological distress: Findings from the Dutch prospective Amsterdam Born Children and their Development study Click here for additional data file. Supplemental material, sj-docx-1-whe-10.1177_17455057231213737 for Carrying an unintended pregnancy to term and long-term maternal psychological distress: Findings from the Dutch prospective Amsterdam Born Children and their Development study by Wieke Y Beumer, Tessa J Roseboom, Marjette H Koot, Tanja Vrijkotte and Jenneke van Ditzhuijzen in Women’s Health

sj-docx-2-whe-10.1177_17455057231213737 – Supplemental material for Carrying an unintended pregnancy to term and long-term maternal psychological distress: Findings from the Dutch prospective Amsterdam Born Children and their Development study Click here for additional data file. Supplemental material, sj-docx-2-whe-10.1177_17455057231213737 for Carrying an unintended pregnancy to term and long-term maternal psychological distress: Findings from the Dutch prospective Amsterdam Born Children and their Development study by Wieke Y Beumer, Tessa J Roseboom, Marjette H Koot, Tanja Vrijkotte and Jenneke van Ditzhuijzen in Women’s Health

## Declarations

**Ethics approval and consent to participate:** The Central Committee on Research Involving Human Subjects in the Netherlands and the medical ethics research committee of the Amsterdam Medical Center, The Netherlands (Phase 1 and 2: MEC 02/039, March 2002; Phase 3: 02/039 # 07.17.1039; and Phase 4: 2015_154#B2015655a) have approved the study protocol. Written informed consent to participate in this study was provided by all participants. Data were processed anonymously.

**Consent for publication:** Written informed consent for publication was provided by the participants. According to the Dutch Central Committee on Research Involving Human Subjects, respondents aged 16 years and over are capable to sign their own form of consent.

**Availability of data and materials:** The data sets presented in this article are not readily available because the data are not publicly available and were used under licence for this study. Requests to access the data sets should be directed to Tanja Vrijkotte, t.vrijkotte@amsterdamumc.nl.

## References

[1] Bearak JM Popinchalk A Beavin C , et al. Country-specific estimates of unintended pregnancy and abortion incidence: a global comparative analysis of levels in 2015–2019. BMJ Glob Health 2022; 7(3): e007151. 10.1136/bmjgh-2021-007151 PMC8943721 35332057

[2] van Ditzhuijzen J Ten Have M de Graaf R , et al. Correlates of common mental disorders among Dutch women who have had an abortion: a longitudinal cohort study. Perspect Sex Reprod Health 2017; 49(2): 123–131. 28453924 10.1363/psrh.12028

[bibr3-17455057231213737] Steinberg JR McCulloch CE Adler NE. Abortion and mental health: findings from The National Comorbidity Survey-Replication. Obstet Gynecol 2014; 123(2): 263–270. 24402590 10.1097/AOG.0000000000000092 PMC3929105

[4] American Psychological Association. Task force on mental health and abortion. Report of the Task Force on Mental Health and Abortion, APA Task Force, Washington, DC, August 2008.

[5] Herd P Higgins J Sicinski K , et al. The implications of unintended pregnancies for mental health in later life. Am J Public Health 2016; 106(3): 421–429. 26691118 10.2105/AJPH.2015.302973 PMC4815713

[6] Gipson JD Koenig MA Hindin MJ. The effects of unintended pregnancy on infant, child and parental health: a review of the literature. Stud Fam Plan 2008; 39(1): 18–38. 10.1111/j.1728-4465.2008.00148.x 18540521

[7] McCrory C McNally S. The effect of pregnancy intention on maternal prenatal behaviours and parent and child health: results of an Irish cohort study. Paediatr Perinat Epidemiol 2013; 27(2): 208–215. 23374066 10.1111/ppe.12027

[bibr8-17455057231213737] Sasaki N Ikeda M Nishi D. Long-term influence of unintended pregnancy on psychological distress: a large sample retrospective cross-sectional study. Arch Womens Ment Health 2022; 25(6): 1119–1127. 36306037 10.1007/s00737-022-01273-1

[9] Abajobir AA Maravilla JC Alati R , et al. A systematic review and meta-analysis of the association between unintended pregnancy and perinatal depression. J Affect Disord 2016; 192: 56–63. 26707348 10.1016/j.jad.2015.12.008

[10] Christensen AL Stuart EA Perry DF , et al. Unintended pregnancy and perinatal depression trajectories in low-income, high-risk Hispanic immigrants. Prev Sci 2011; 12(3): 289–299. 21537899 10.1007/s11121-011-0213-x PMC3742050

[bibr11-17455057231213737] Abbasi S Chuang CH Dagher R , et al. Unintended pregnancy and postpartum depression among first-time mothers. J Womens Health 2013; 22(5): 412–416. 10.1089/jwh.2012.3926 PMC3701312 23488527

[12] Mercier RJ Garrett J Thorp J , et al. Pregnancy intention and postpartum depression: secondary data analysis from a prospective cohort. BJOG 2013; 120(9): 1116–1122. 23651010 10.1111/1471-0528.12255 PMC3708972

[13] Hill B Kothe EJ Currie S , et al. A systematic mapping review of the associations between pregnancy intentions and health-related lifestyle behaviours or psychological wellbeing. Prev Med Rep 2019; 14: 100869. 31011520 10.1016/j.pmedr.2019.100869 PMC6465583

[14] Foster DG Steinberg JR Roberts SC , et al. A comparison of depression and anxiety symptom trajectories between women who had an abortion and women denied one. Psychol Med 2015; 45(10): 2073–2082. 25628123 10.1017/S0033291714003213 PMC5004731

[15] Schmiege S Russo NF. Depression and unwanted first pregnancy: longitudinal cohort study. BMJ 2005; 331(7528): 1–5. 16257993 10.1136/bmj.38623.532384.55 PMC1298850

[16] Harris LF Roberts SC Biggs MA , et al. Perceived stress and emotional social support among women who are denied or receive abortions in the United States: a prospective cohort study. BMC Womens Health 2014; 14(76): 1–11. 24946971 10.1186/1472-6874-14-76 PMC4080695

[17] Need A Ultee W Levels M , et al. Meningen over abortus in West-Europa, 1981–2000. Mens en Maatsch 2008; 83: 1–22.

[18] Muis Q Sieben I Reeskens T , et al. Seksueel-ethische permissiviteit: trends in Nederland 1981–2017. Mens en Maatsch 2019; 94(4): 429–458.

[19] Maxson P Miranda ML. Pregnancy intention, demographic differences, and psychosocial health. J Womens Health 2011; 20(8): 1215–1223. 10.1089/jwh.2010.2379 21671765

[bibr20-17455057231213737] Finer LB Henshaw SK. Disparities in rates of unintended pregnancy in the United States, 1994 and 2001. Perspect Sex Reprod Health 2006; 38(2): 90–96. 16772190 10.1363/psrh.38.090.06

[21] Ikamari L Izugbara C Ochako R . Prevalence and determinants of unintended pregnancy among women in Nairobi, Kenya. BMC Pregnancy Childbirth 2013(19): 13–69. 10.1186/1471-2393-13-69 PMC3607892 23510090

[22] De Graaf R Ten Have M Van Dorsselaer S . De psychische gezondheid van de Nederlandse bevolking: NEMESIS-2: opzet en eerste resultaten. Report, Trimbos, Utrecht, November 2010.

[23] van der Heide I Wang J Droomers M , et al. The relationship between health, education, and health literacy: results from the Dutch adult literacy and life skills survey. J Health Commun 2013; 18(Suppl. 1): 172–184. 24093354 10.1080/10810730.2013.825668 PMC3814618

[24] Steinberg JR Tschann JM. Childhood adversities and subsequent risk of one or multiple abortions. Soc Sci Med 2013; 81: 53–59. 23312795 10.1016/j.socscimed.2012.11.011 PMC3699177

[25] Boden JM Fergusson DM Horwood LJ. Experience of sexual abuse in childhood and abortion in adolescence and early adulthood. Child Abuse Negl 2009; 33(12): 870–876. 19897244 10.1016/j.chiabu.2009.04.006

[26] Santelli JS Lindberg LD Orr MG , et al. Toward a multidimensional measure of pregnancy intentions evidence from the United States. Stud Fam Plann 2009; 40(2): 87–100. 19662801 10.1111/j.1728-4465.2009.00192.x

[27] Klerman LV. The intendedness of a pregnancy: a concept in transition. Matern Child Health J 2000; 4(3): 155–162. 11097502 10.1023/a:1009534612388

[28] Barrett G Smith SC Wellings K. Conceptualisation, development, and evaluation of a measure of unplanned pregnancy. J Epidemiol Community Health 2004; 58(5): 426–433. 15082745 10.1136/jech.2003.014787 PMC1732751

[29] Aiken AR Dillaway C Mevs-Korff N. A blessing I can’t afford: factors underlying the paradox of happiness about unintended pregnancy. Soc Sci Med 2015; 132: 149–155. 25813729 10.1016/j.socscimed.2015.03.038 PMC4400251

[30] van Eijsden M Vrijkotte TG Gemke RJ , et al. Cohort profile: the Amsterdam born children and their development (ABCD) study. Int J Epidemiol 2011; 40(5): 1176–1186. 20813863 10.1093/ije/dyq128

[31] Alderliesten ME Vrijkotte TG van der Wal MF , et al. Late start of antenatal care among ethnic minorities in a large cohort of pregnant women. BJOG 2007; 114(10): 1232–1239. 17655734 10.1111/j.1471-0528.2007.01438.x

[32] Tromp M van Eijsden M Ravelli AC , et al. Anonymous non-response analysis in the ABCD cohort study enabled by probabilistic record linkage. Paediatr Perinat Epidemiol 2009; 23(3): 264–272. 19775388 10.1111/j.1365-3016.2009.01030.x

[33] Preacher KJ Coffman DL . Computing power and minimum sample size for RMSEA, https://www.quantpsy.org/rmsea/rmsea.htm (2006, accessed on 20 May 2023).

[34] MacCallum RC Browne MW Sugawara HM. Power analysis and determination of sample size for covariance structure modeling. Psychol Methods 1996; 1(2): 130–149.

[35] Krumpal I. Determinants of social desirability bias in sensitive surveys: a literature review. Qual Quant 2011; 47(4): 2025–2047.

[36] Spielberger CD. State-Trait Anxiety Inventory for Adults (STAI-AD) (APA Psyc Tests). Washington, DC: American Psychological Association, 1983.

[37] Van der Ploeg HM . Validity of the Zelf-Beoordelings-Vragenlijst (A Dutch version of the Spielberger State-Trait Anxiety Inventory). Ned Tijdschr Psychol 1980; 35(4): 243–249.

[38] Radloff LS. The CES-D scale: a self-report depression scale for research in the general population. Appl Psychol Meas 1977; 1(3): 385–401.

[39] Zhang B Fokkema M Cuijpers P , et al. Measurement invariance of the center for epidemiological studies depression scale (CES-D) among Chinese and Dutch elderly. BMC Med Res Methodol 2011; 11: 74. 21595982 10.1186/1471-2288-11-74 PMC3123656

[40] De Beurs E . De DASS: een vragenlijst voor het meten van despressie, angst en stress. Gedragstherapie 2001; 34: 35–53.

[41] Lovibond PF Lovibond SH. The structure of negative emotional states: comparison of the Depression Anxiety Stress Scales (DASS). Behav Res Ther 1995; 33(3): 335–343. 7726811 10.1016/0005-7967(94)00075-u

[42] Evans L Haeberlein K Chang A , et al. Convergent validity and preliminary cut-off scores for the anxiety and depression subscales of the DASS-21 in US adolescents. Child Psychiatry Hum Dev 2021; 52(4): 579–585. 32816139 10.1007/s10578-020-01050-0

[43] Coker AO Coker OO Sanni D. Psychometric properties of the 21-item Depression Anxiety Stress Scale (DASS-21). Afr Res Rev 2018; 12(2): 135–142.

[44] Carleton RN Thibodeau MA Teale MJ , et al. The center for epidemiologic studies depression scale: a review with a theoretical and empirical examination of item content and factor structure. PLoS ONE 2013; 8(3): e58067. 10.1371/journal.pone.0058067 PMC3585724 23469262

[45] American Psychological Association. Diagnostic and statistical manual of mental disorders. 5th ed. Washington, DC: American Psychiatric Publishing, 2013.

[46] Vautier S. A longitudinal SEM approach to STAI data: two comprehensive multitrait-multistate models. J Pers Assess 2004; 83(2): 167–179. 15456653 10.1207/s15327752jpa8302_11

[47] Vigneau F Cormier S. The factor structure of the State-Trait Anxiety Inventory: an alternative view. J Pers Assess 2008; 90(3): 280–285. 18444124 10.1080/00223890701885027

[48] RStudio Team. RStudio: integrated development for R. Boston, MA: RStudio Team, 2020.

[49] Schafer JL Graham JW. Missing data: our view of the state of the art. Psychol Methods 2002; 7(2): 147–177. 12090408

[50] Lee T Shi D. A comparison of full information maximum likelihood and multiple imputation in structural equation modeling with missing data. Psychol Methods 2021; 26(4): 466–485. 33507765 10.1037/met0000381

[51] Rosseel Y. Lavaan: an R package for structural equation modeling. J Stat Softw 2012; 48(2): 1–36.

[52] Ferguson CJ. An effect size primer: a guide for clinicians and researchers. Prof Psychol Res Pr 2009; 40(5): 532–538.

[53] Lai K Green SB. The problem with having two watches: assessment of fit when RMSEA and CFI disagree. Multivariate Behav Res 2016; 51(2–3): 220–239. 27014948 10.1080/00273171.2015.1134306

[bibr54-17455057231213737] Yu CY. Evaluating cutoff criteria of model fit indices for latent variable models with binary and continuous outcomes. PhD Thesis, University of California, Oakland, CA, 2002.

[bibr55-17455057231213737] Browne MW Cudeck R. Alternative ways of assessing model fit. Sociol Methods Res 1992; 21: 230–258.

[56] Bentler PM Bonett DG. Significance tests and goodness of fit in the analysis of covariance structures. Psychol Bull 1980; 88(3): 588–606.

[57] Sass DA Schmitt TA Marsh HW. Evaluating model fit with ordered categorical data within a measurement invariance framework: a comparison of estimators. Struct Equ Modeling 2014; 21(2): 167–180.

[58] Cohen J. Statistical power analysis for the behavioral sciences. 2nd ed. New York: Routledge, 1988.

[59] Cheng D Schwarz EB Douglas E , et al. Unintended pregnancy and associated maternal preconception, prenatal and postpartum behaviors. Contraception 2009; 79(3): 194–198. 19185672 10.1016/j.contraception.2008.09.009

[bibr60-17455057231213737] Barton K Redshaw M Quigley MA , et al. Unplanned pregnancy and subsequent psychological distress in partnered women: a cross-sectional study of the role of relationship quality and wider social support. BMC Pregnancy Childbirth 2017; 17(1): 44. 28122585 10.1186/s12884-017-1223-x PMC5267424

[61] Currao A Mezuk B. Association between unintended pregnancy and internalizing disorders among Latina and Asian American mothers. J Affect Disord 2019; 258: 117–124. 31401539 10.1016/j.jad.2019.07.068

[62] Najman JM Morrison J Williams G , et al. The mental health of women 6 months after they give birth to an unwanted baby: a longitudinal study. Soc Sci Med 1991; 32(3): 241–247. 2024133 10.1016/0277-9536(91)90100-q

[63] Wellings K Jones KG Mercer CH , et al. The prevalence of unplanned pregnancy and associated factors in Britain: findings from the third National Survey of Sexual Attitudes and Lifestyles (Natsal-3). Lancet 2013; 382(9907): 1807–1816. 24286786 10.1016/S0140-6736(13)62071-1 PMC3898922

[64] Rocca CH Wilson MR Jeon M , et al. Stability of retrospective pregnancy intention reporting among women with unwanted pregnancies in the United States. Matern Child Health J 2019; 23(11): 1547–1555. 31236825 10.1007/s10995-019-02782-9 PMC6786959

[bibr65-17455057231213737] Moreau C Bohet A Le Guen M , et al. Unplanned or unwanted: a randomized study of national estimates of pregnancy intentions. Fertil Sterel 2014; 102(6): 1663–1670. 10.1016/j.fertnstert.2014.08.011 25241373

[66] Saleem HT Surkan PJ. Parental pregnancy wantedness and child social-emotional development. Matern Child Health J 2014; 18(4): 930–938. 23793490 10.1007/s10995-013-1320-z PMC4437702

[67] Tyrlik M Konecny S Kukla L. Predictors of pregnancy-related emotions. J Clin Med Res 2013; 5(2): 112–120. 23518672 10.4021/jocmr1246e PMC3601497

[68] Walker AL de Rooij SR Dimitrova MV , et al. Psychosocial and peripartum determinants of postpartum depression: findings from a prospective population-based cohort. Compr Psychiatry 2021; 108: 152239. 33905988 10.1016/j.comppsych.2021.152239

[69] Auerbach SL Coleman-Minahan K Alspaugh A , et al. Critiquing the unintended pregnancy framework. J Midwifery Womens Health 2023; 68(2): 170–178. 36637112 10.1111/jmwh.13457

[70] Wolke D Waylen A Samara M , et al. Selective drop-out in longitudinal studies and non-biased prediction of behaviour disorders. Br J Psychiatry 2009; 195(3): 249–256. 19721116 10.1192/bjp.bp.108.053751 PMC2802508

[71] Jakobsen JC Gluud C Wetterslev J , et al. When and how should multiple imputation be used for handling missing data in randomised clinical trials – a practical guide with flowcharts. BMC Med Res Methodol 2017; 17(162): 1–10. 29207961 10.1186/s12874-017-0442-1 PMC5717805

[72] Leigh B Milgrom J. Risk factors for antenatal depression, postnatal depression and parenting stress. BMC Psychiatry 2008; 8: 24. 18412979 10.1186/1471-244X-8-24 PMC2375874

[73] Lovibond PF. Long-term stability of depression, anxiety, and stress syndromes. J Abnorm Psychol 1998; 107(3): 520–526. 9715586 10.1037//0021-843x.107.3.520

[74] Rocca CH Smith MG Hale NL , et al. Ranges of pregnancy preferences and contraceptive use: results from a population-based survey in the southeast United States. Perspect Sex Reprod Health 2022; 54(3): 90–98. 36071572 10.1363/psrh.12205

[75] Bronte-Tinkew J Scott ME Horowitz A , et al. Pregnancy intentions during the transition to parenthood and links to coparenting for first-time fathers of infants. Parenting 2009; 9(1–2): 1–35.

[76] Waller MR Bitler MP. The link between couples’ pregnancy intentions and behavior: does it matter who is asked. Perspect Sex Reprod Health 2008; 40(4): 194–201. 19067932 10.1363/4019408 PMC5751747

