# Supplementary material for: Carrying an unintended pregnancy to term and long-term maternal psychological distress: Findings from the Dutch prospective Amsterdam Born Children and their Development study
Source: Womens Health (Lond). 2023 Dec 7;19:17455057231213737. doi: 10.1177/17455057231213737 (PMC10704944; doi:10.1177/17455057231213737)
Supplement: sj-docx-1-whe-10.1177_17455057231213737 – Supplemental material for Carrying an unintended pregnancy to term and long-term maternal psychological distress: Findings from the Dutch prospective Amsterdam Born Children and their Development study [file sj-docx-1-whe-10.1177_17455057231213737.docx]

# Supplementary file: Questionnaires used

Manuscript title: **Carrying unintended pregnancy to term and long-term maternal psychological distress. Findings from the Dutch prospective ABCD study**

## Questionnaires to measure psychological distress (outcome)

### Antepartum & around 3 months postpartum

- **Depression:** Center for Epidemiological Studies Depression scale (CES-D) (Radloff, 1977; Zhang et al., 2011)
- **Anxiety:** State-Trait Anxiety Inventory State form (STAI-S) (Spielberger, 1983; Van der Ploeg, 1980)

### 5-6 and 11-12 years postpartum

- Depression Anxiety Stress Scale (DASS-21) (De Beurs, 2001; Lovibond & Lovibond, 1995)

## Pregnancy intentions (predictor)

1. ‘*I did not want to be pregnant (anymore*)’ (unwantedness)
2. ‘*This pregnancy happened too soon*’ (mistiming)
3. *‘I am happy to be pregnant’* (unhappiness; recoded)

Items were rated on a 4-point Likert scale ranging from 0 (definitely not true) to 3 (very true), with a higher score indicating more unintendedness.

## Controls

These questions were formulated and tested by researchers from the Dutch ABCD study (Van Eijsden et al., 2011). Questions were asked about

1. antepartum maternal age;
2. ethnicity (being born in the Netherlands; yes/no);
3. educational level (measured in years of educational attainment after primary school);
4. having a paid job (yes/no) ;
5. being a single parent during pregnancy (yes/no);
6. experiences with sexual assault (yes/no);
7. experiences with physical assault (yes/no);
8. sex of the child (girl/boy);
9. number of children already present in the family.

## References

- De Beurs, E. (2001). De DASS: een vragenlijst voor het meten van despressie, angst en stress.
- Lovibond, P. F., & Lovibond, S. H. (1995). The structure of negative emotional states: comparison of the Depression Anxiety Stress Scales (DASS). *Behav Res Ther*, *33*(3), 335-343. <https://doi.org/10.1016/0005-7967(94)00075-u>
- Radloff, L. S. (1977). The CES-D scale: a self-report depression scale for research in the general population. *Applied Psychological Measurement*, *1*(3), 385-401.
- Spielberger, C. D. (1983). State-trait anxiety inventory for adults.
- Van der Ploeg, H. M. (1980). Validity of the Zelf-Beoordelings-Vragenlijst (A Dutch version of the Spielberger State-Trait Anxiety Inventory). *Nederlands Tijdschrift voor de Psychologie en Haar Grensgebieden*, *35*(4), 243-249.
- Van Eijsden, M., Vrijkotte, T. G., Gemke, R. J., & Van der Wal, M. F. (2011). Cohort profile: the Amsterdam Born Children and their Development (ABCD) study. *International Journal of Epidemiology*, *40*(5), 1176-1186. <https://doi.org/10.1093/ije/dyq128>
- Zhang, B., Fokkema, M., Cuijpers, P., Juan, L., Smits, N., & Beekman, A. (2011). Measurement invariance of the center for epidemiological studies depression scale (CES-D) among Chinese and Dutch elderly. *BMC Medical Research Methodology*, *11*(74). <https://doi.org/https://doi.org/10.1186/1471-2288-11-74>
